# Acute and chronic phase myocardial tissue characteristics in non ST-elevation myocardial infarction in dependence of early versus delayed revascularisation assessed by cardiac magnetic resonance imaging

**DOI:** 10.1186/1532-429X-16-S1-P185

**Published:** 2014-01-16

**Authors:** Dominik Buckert, Vinzenz Hombach, Volker Rasche, Wolfgang Rottbauer, Peter Bernhardt

**Affiliations:** 1 University of Ulm, Ulm, Germany

## Background

According to current guidelines, an adequate timeframe for revascularisation in the setting of acute non-ST-elevation myocardial infarction (NSTEMI) is considered by current guidelines as being up to 72 hours after symptom onset. Soon after myocardial infarction, changes take place in the affected myocardium that can be visualized and measured by cardiac magnetic resonance imaging (CMR). Aim of the present study was to compare tissue characteristics in patients with NSTEMI that underwent early vs. delayed revascularisation. Therefore, myocardial tissue characteristics were evaluated in the acute and chronic phase of reperfused NSTEMI patients and the findings were correlated to the particular symptom-to-reperfusion time.

## Methods

In 53 patients presenting with NSTEMI, CMR imaging was performed in addition to invasive coronary angiography. The patients were examined on a 1.5 T whole-body scanner using a 32-channel phased-array surface coil. Left-ventricular volumes were assessed by a standard steady-state free-precession sequence. Myocardial edema was evaluated using a 3D T2-weighted black-blood fat-saturated spin-echo sequence. Microvascular obstruction and late gadolinium enhancement were measured by a 3D phase-sensitive inversion-recovery gradient echo sequence about 12 minutes after intravenous administration of gadolinium based contrast agent. CMR imaging was conducted shortly after coronary revascularisation and about 90 days later at follow-up. Results were gained by two experienced readers in consensus.

## Results

Patients with early (< 12 hours) vs. delayed (> 12 hours) symptom-to-reperfusion time did not differ significantly concerning CMR characteristics at baseline or follow up. In all patients left ventricular myocardial mass (p < 0.0001), infarct size (p = 0.007) and microvascular obstruction (p = 0.02) were significantly reduced at the follow-up in comparison to baseline. In patients with observed microvascular obstruction, infarct size was significantly larger (p = 0.003). Infarct size was negative correlated to the myocardial salvage index (R = -0.6428, p < 0.0001).

## Conclusions

Myocardial tissue characteristics in patients with NSTEMI in the acute and chronic phase can be assessed by CMR imaging. There were no significant differences in patients with early in comparison to delayed revascularisation. Based on these results, CMR-based parameters in NSTEMI patients may serve as a surrogate endpoint in therapeutic studies.

## Funding

This study was partially funded by a research grant of the German Heart Research Foundation (F/02/10).

